# A Viable Population of the European Red Squirrel in an Urban Park

**DOI:** 10.1371/journal.pone.0105111

**Published:** 2014-08-15

**Authors:** Célia Rézouki, Anne Dozières, Christie Le Cœur, Sophie Thibault, Benoît Pisanu, Jean-Louis Chapuis, Emmanuelle Baudry

**Affiliations:** 1 Centre d'Ecologie et des Sciences de la Conservation, Muséum National d'Histoire Naturelle, Paris, France; 2 Laboratoire Ecologie Systématique et Evolution, Université Paris-Sud, Orsay, France; University of Calgary, Canada

## Abstract

Whether urban parks can maintain viable and self-sustaining populations over the long term is questionable. In highly urbanized landscapes, urban parks could play a role in biodiversity conservation by providing habitat and resources to native species. However, populations inhabiting urban parks are usually small and isolated, leading to increased demographic stochasticity and genetic drift, with expected negative consequences on their viability. Here, we investigated a European red squirrel population located in an urban park close to Paris, France (Parc de Sceaux; 184 ha) to assess its viability. Using mitochondrial D-loop sequences and 13 microsatellite loci, we showed that the population presented high levels of genetic variation and no evidence of inbreeding. The size of the population was estimated at 100–120 individuals based on the comparison of two census techniques, Distance Sampling and Capture-Mark-Recapture. The estimated heterozygosity level and population size were integrated in a Population Viability Analysis to project the likelihood of the population's persistence over time. Results indicate that the red squirrel population of this urban park can be viable on the long term (i.e. 20 years) for a range of realistic demographic parameters (juvenile survival at least >40%) and immigration rates (at least one immigration event every two years). This study highlights that urban parks can be potential suitable refuges for the red squirrel, a locally threatened species across western European countries, provided that ecological corridors are maintained.

## Introduction

Urbanization is intensifying worldwide, generating loss and fragmentation of wildlife habitats [1]. These alterations are responsible for local extinctions of native species in urban and suburban environments [2] and have become a major concern for wildlife conservation [3]. In highly urbanized landscapes, green parks could play an important role in biodiversity conservation by providing habitat and resources to native species [4], [5].

However, populations inhabiting urban parks are usually relatively small and isolated from other populations by the urban matrix, which has demographic and genetics consequences [6]. First, small and isolated populations undergo increased demographic stochasticity and reduced recolonization from other populations (rescue effects), increasing their extinction risk [7]. Additionally, small and isolated population may suffer from inbreeding depression, accumulation of deleterious mutations and reduced rate of fixation of beneficial mutations, which can further increase their extinction risks [8]. Consequently, it is questionable whether urban parks can maintain populations that are viable and self-sustaining over the long term.

The Eurasian red squirrel, *Sciurus vulgaris* (L.), is a well known arboreal rodent which prefers mixed coniferous and deciduous forests across Northern Europe [9]. It is widespread and currently common throughout much of its range, although locally vulnerable and in decline [10], resulting in its classification as a protected species in Europe since 1979. It is especially threatened by the competition with the introduced grey squirrel *Sciurus carolinensis* [11], [12], currently present in Britain, Ireland and Italy, where it gradually reaches the Franco-Italian border. In these countries, the grey squirrel outcompetes the red squirrel for resources in woodlands, leading to a significant decline in native red squirrel populations, and its complete replacement over large areas in Britain [11], [12]. The red squirrel appears to be very sensitive to forest fragmentation and its presence is determined by the degree of isolation, the size and the habitat quality of the forest fragments [13]–[15]. *S. vulgaris* seems however to be well adapted to life in urban environments as it readily inhabits parks, gardens and suburban forests [16]–[18], where it sometimes reaches higher densities than in natural environments [18], [19].

The aim of the present study was to assess if a red squirrel population inhabiting an urban park could be viable, i.e. self-sustaining over the long term. Firstly, genetic variability levels were examined with mitochondrial and nuclear markers and compared to those of a control population located in an unfragmented natural habitat of the French Alps. Secondly, population size was estimated by comparing the results of two different census techniques, Distance Sampling and Capture-Mark-Recapture. Finally, the estimated heterozygosity level and population size were integrated in a Population Viability Analysis (PVA) [20]–[22] to project the likelihood of the population's persistence over time.

## Materials and Methods

### Study sites and sampling collection

**Figure 1:**
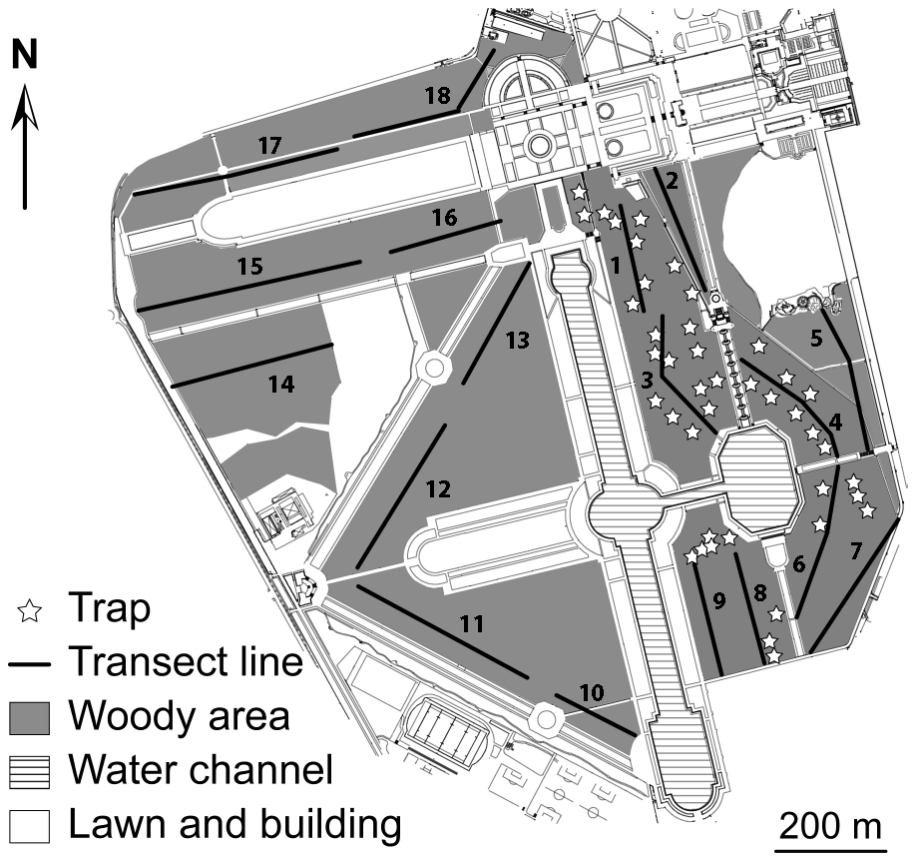
Map of the Parc de Sceaux showing the distribution of line transects, traps, and woody areas suitable to red squirrels.

The Parc de Sceaux (184 ha; 48°46′4″N, 2°17′55″E) is located 9 km south-west from Paris. It is a highly frequented park where the public actively feeds animals, in particular red squirrels. The park is mainly composed of open habitats formed by lawns, meadows, French gardens, and man-made water channels (112 ha) (Fig. 1). Closed habitats are made of woods dominated by deciduous trees (dominant: *Acer* spp., *Fraxinus excelsior*; rarest: *Carpinus betulus*, *Quercus* spp.). The eastern part of the park (28 ha) is covered by mature woodlands providing good quality habitats for the red squirrel and includes partially protected areas. The western part (44 ha) is composed of younger woodlots with exotic trees and provides poorer quality habitats for the red squirrel. An urban matrix isolates the park from other forest patches, the nearest being located approximately 1 km west. Between 2010 and 2013, the red squirrel population has been monitored biannually (February and June) using a Capture-Mark-Recapture procedure (CMR) in the protected areas of the eastern part of the park [23] (Fig. 1). A total of 68 squirrels were captured. For each individual, a hair sample was taken and stored in 95% ethanol.

The control population used for the genetic analyses was located in a large unfragmented forest (the Massif de l'Epine, Savoie, 45°36′3″N, 5°49′3″E) in the French Alps. The habitat was mainly composed of mixed woodlands dominated by spruces (*Picea abies*), firs (*Abies* spp.), and beeches (*Fagus sylvatica*). In this population, 23 individuals were sampled between 2009 and 2011, over six mark-recapture sessions [24].

### Genetic analyses

Because red squirrels are protected, we used a non- invasive method for obtaining DNA samples. DNA was extracted from hair samples using a Macherey-Nagel NucleoSpin Tissue Kit, following the manufacturer's protocol. Mitochondrial DNA variation was assayed by the amplification of a 486 bp fragment of the D-loop using the procedure described in [19]. DNA was then sequenced with the PCR primers, using standard Sanger sequencing techniques. Mitochondrial sequences were examined and edited using ProSeq 3.5 [25]. Sequences were deposited into the GenBank database under accession numbers KM030177 to KM030254. We used DNAsp5 to analyze sequence variations.

Thirteen microsatellites loci were used to assay nuclear genetic variation (File S1). The size of the microsatellite alleles was determined using GeneMapper 4.0 (Applied Biosystems). The microsatallite genotypes are presented in the File S1. Microsatellite diversity was estimated using FSTAT 2.9.3.2 [26]. Arlequin 3.11 [27] was used to calculate the observed (Ho) and expected heterozygosity (He), and to test for departures from Hardy-Weinberg equilibrium.

The Bayesian clustering method implemented in structure v. 2.3.4 [28] was used to investigate population structure and the presence of potential migrants in the Sceaux population. To determine the most likely number of genetic groups in our sampling, we first used the admixture model, where each individual draws some fraction of its genome from each of the genetic groups. We then used the USEPOPINFO model, where the sampling location (here the Parc de Sceaux) is used to test whether any individuals in the sample are immigrants. Each run of the models was based on 1 000 000 MCMC simulations with an initial ‘burn-in' period of 250 000; 10 iterations were run for each K value ranging from 1 to 3.

### Population size estimate

Distance sampling [29] from line-transects counts (Fig. 1) was used to estimate the population density throughout the park and investigate possible differences between the eastern and western parts (File S2), which differ in habitat quality. Results were compared to density estimates inferred from mark-recapture monitoring using baited live-trapping (File S2), which could only be carried out in the protected areas of the eastern part of the park (Fig. 1). If the results obtained by the two methods are similar, density estimates from distance sampling will be used to calculate the absolute population size of red squirrels in the park, taking into account the area of suitable habitat.

### Population Viability Analyses (PVA)

The viability of the population was investigated using the stochastic population modeling program Vortex 9.99 [30]. Demographic parameters used to set the PVA models were taken from the literature, or were estimated for our study population. Based on data collected in the literature, we set the mating system of the red squirrel to polygynous [9] and assumed that 35% of the breeding females breed twice in a year [31], [32], i.e., they have a spring and a summer litter [31], [9]. Models were parameterized with a mean litter size of 3 and a maximum of 6 offspring, and a sex ratio of 1∶1 [33], [9]. Based on monitoring data of the Sceaux population, adult annual survival rates were estimated to be 68% (±12) for females and 63% (±13) for males (A. Dozières, pers. com.) using the Pollock's robust design method [34]. Finally, different values, from pessimistic to optimistic, were set for demographic parameters for which no data were available (age at first litter and juvenile survival) and for the annual percentage of breeding female at high density, for which we obtained different estimates in different years (File S3). For each parameter combination, or scenario, we first investigated the general deterministic trends of population dynamics by calculating the asymptotic growth rate (λ). Then, we incorporated environmental and demographic stochasticity and run each scenario 1000 times over 20 years, to determine the stochastic growth rate and the extinction probability under stochastic fluctuations. The limit was set to 20 years which represents about 10 generations for red squirrels and corresponds to the timeframe for defining a population as endangered according to its probability of extinction [35].

Finally, the observed allelic frequencies of the polymorphic microsatellite loci were incorporated in the structure of the starting population to project the evolution of the heterozygosity rate over time as a function of immigration rate into the park. Several scenarios were simulated to test the effect of a total isolation on the population genetic variability, and to assess which immigration rate would be needed in the population to retain genetic diversity. To generate these immigration events, a source population with a stable size of 100 individuals was artificially created and the effect of juvenile and subadult (from 1 to 2 years) immigration events were tested, by varying the annual probabilities of dispersal from one immigrant every five years to five immigrants per year.

### Ethics statement

All conducted experiments complied with the current laws of France. In this study we obtained authorizations to transport corpses (e.g. [19]), and capture-mark individuals of this protected species. This derogation was authorized by the French Ministry of Ecology, Sustainable Development and Energy (MEDDE: authorization n°08/550) and by the ethics committee Cuvier (n°68–011). No specific permissions were required for accessing field monitoring sites.

## Results

### Genetic analyses

Sequences of mtDNA D-loop were successfully determined for 65 individuals from the Parc de Sceaux population and 13 from the Savoie population. The two populations displayed 3 and 6 haplotypes respectively. Haplotype diversities were similar in the two populations (0.60±0.03 and 0.72±0.13 in the Parc de Sceaux and Savoie populations respectively), as were nucleotide diversities (0.0093±0.0005 and 0.0099±0.0016 in the Sceaux and Savoie populations respectively). The relatively low level of amplification success (85.7% for mtDNA and 74.5% for microsatellite, see below) is probably due to the low DNA content of the hair samples and is well within the range of what is typically obtained with non-invasive sampling techniques [36].

All microsatellite loci were polymorphic except the Scv15 locus in the Parc de Sceaux population and the Scv14 locus in the population of Savoie. The total number of alleles per polymorphic locus varied from 1 to 10 for both populations. Values of observed and expected heterozygosity are given for each locus in Table 1. The mean values of these genetic diversity indices were similar between the two populations. No significant departures from Hardy-Weinberg equilibrium were recorded at 12 of 13 other loci, suggesting that both populations are at Hardy-Weinberg equilibrium, and that their inbreeding levels are therefore low, although that does not exclude that a few individuals originated from related parents. A significant departure from Hardy-Weinberg equilibrium was recorded at the Scv3 locus in both populations (Table 1), indicating the likely presence of a null allele at this locus.

**Table 1:** Results of the genetic diversity analyses obtained per locus for the red squirrel populations.

Locus	N. genotyped	Heterozygosity		*P*_Hardy-Weinberg_
		observed	expected	
**Parc de Sceaux**				
Rsu3	61	0.393	0.465	0.269
Rsu4	48	0.521	0.462	0.717
Rsu5	38	0.474	0.417	0.459
Rsu6	65	0.554	0.526	0.021
Scv6	54	0.704	0.592	0.322
Scv8	47	0.255	0.232	1.000
Scv1	64	0.500	0.395	0.118
Scv12	55	0.255	0.230	1.000
Scv13	54	0.426	0.408	0.178
Scv14	64	0.516	0.627	0.159
Scv15	52	-	-	-
Scv3	59	0.678	0.822	*0.001
Scv9	46	0.522	0.518	1.000
*Mean*	*-*	*0.483*	*0.475*	*-*
**Massif de l'Epine**				
Rsu3	20	0.450	0.522	0.789
Rsu4	8	0.375	0.442	0.379
Rsu5	10	0.100	0.100	1.000
Rsu6	21	0.190	0.251	0.339
Scv6	15	0.800	0.632	0.139
Scv8	10	0.300	0.279	1.000
Scv1	9	0.889	0.725	1.000
Scv12	11	0.364	0.645	0.140
Scv13	9	0.444	0.451	0.247
Scv14	19	-	-	-
Scv15	18	0.111	0.110	1.000
Scv3	18	0.778	0.873	*0.000
Scv9	6	0.833	0.712	0.340
*Mean*	*-*	*0.470*	*0.479*	*-*

The Bayesian clustering method implemented in Structure was used to infer K, the most likely number of genetic groups in the Sceaux population. Comparisons of the log-likelihood of the different runs for the different K tested (between 1 and 3) revealed a clear peak at K = 1 (−*LL* = 1143.0, 1186.2 and 1300.5 for K = 1, 2 and 3 respectively). We found that all sampled individuals had a posterior probability higher than 0.915 to be correctly assigned to the Sceaux population, suggesting that none of them were immigrants from another genetically distinct population.

### Population size

Considering all visual surveys performed along transects, a total of 169 sightings were recorded during the distance sampling sessions. Examination of the histogram of perpendicular sighting distances did not indicate any apparent signs of movement before detection, and the narrow shoulder emerging indicated that detection falls rapidly with distance (Fig. S1). The Hazard-rate function with cosine adjustment was selected as the best model (Table S1). In the eastern part of the park, density ranged between 2.4±0.9 in October and 2.3±0.7 individuals per ha in March whereas in the western part, densities were estimated at 1.2±0.5 and 0.9±0.4 individuals per ha respectively (Table 2). For density estimation using spatially explicit capture-recapture, the best model included a learned response after the first capture of an individual on both capture probability and movement scale (Table S2). With this model, densities were estimated in the eastern part of the park at 3.0±0.8 in October and 2.6±0.7 individuals per ha in February, which were close to those inferred from distance sampling (Table 2).

**Table 2:** Estimates of red squirrel density (individuals per ha) using distance sampling in two areas of the Parc de Sceaux and spatially explicit capture-recapture models in the eastern area.

Method	Area	Months	Counts	Density±SE	95%C.I.
Distance sampling	Western	November-December 2012	35	1.2±0.5	0.5–3.0
	Eastern		56	2.4±0.9	1.1–5.5
	Western	March 2013	24	0.9±0.4	0.3–2.3
	Eastern		54	2.3±0.7	1.3–4.4
Capture-Recapture	Eastern	October 2012	-	3.0±0.8	1.8–5.0
	Eastern	February 2013	-	2.6±0.7	1.6–4.3

Densities derived from the distance sampling estimations in each side of the park were multiplied by their corresponding surface, leading to an estimation of 124 (95%CI: 53–289) individuals for the October-November session and 104 (95%CI: 50–227) individuals for the March session, with quite similar but large confidence intervals.

### Population Viability Analysis (PVA)

**Figure 2:**
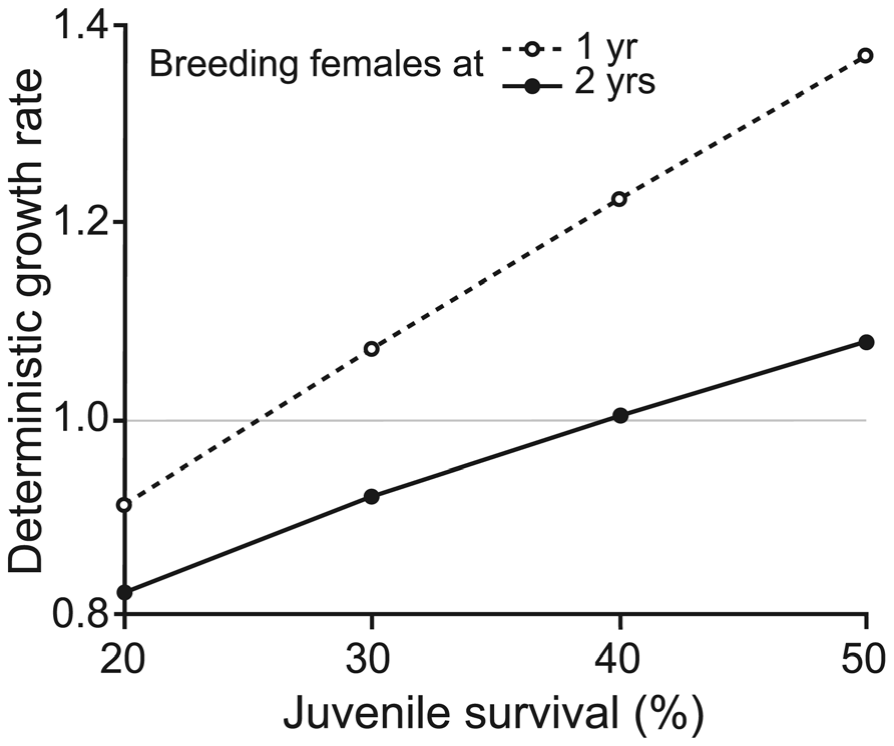
Relationships between the deterministic growth rate of the population, the rate of juvenile survival and the age at which females have their first litter.

Deterministic model predicted that the population would be intrinsically growing (asymptotic growth rate λ>1) only if the juvenile survival exceeds 25% when individuals have their first litter at one year-old, and if it exceeds 40% for a mean age at first litter of 2 years-old (Figure 2). If those conditions are not met, the population is declining and is therefore doomed to extinction.

However, populations with demographic parameters that allow an intrinsic growth did not necessarily exhibit a positive growth rate under demographic and environmental stochasticity. Under stochastic events, the growth rate ranged from −0.11 (SD 0.51) to 0.14 (SD 0.39) for the most optimistic scenarios with λ≥1 (Table 3). The extinction probability after 20 years of projection was low (PE<0.10) for a juvenile survival of 40% when the age at first litter was set at 1 year-old, and for a juvenile survival and a high-density reproductive rate of 50% when the age at first litter was set at 2 years-old (Table 3). Because the lower boundaries of the confidence intervals of the population size estimates were about 50 animals, we also run PVA simulations with a starting population size of 50 animals while keeping the carrying capacity at 120 individuals. The results showed that, as expected, this increased the probability of extinction for each case (Table S3) but it did not change the main conclusion that the population may be viable with a juvenile survival greater or equal to 40% and an age at first reproduction of 1 year-old.

**Table 3:** Results of simulations run for the population viability analysis of red squirrels population of the Parc de Sceaux.

Scenarios	Stochastic growth rate (SD)	Probability of extinction	Expected heterozygosity (SD)
	Rate (%) of				
Age at first litter	Juvenile survival	Breeding females at high density			
1 year	20	35	−0.24 (0.49)	0.82	0.32 (0.09)
		50	−0.21 (0.55)	0.78	0.32 (0.10)
	30	35	−0.11 (0.51)	0.39	0.33 (0.08)
		50	−0.07 (0.50)	0.34	0.34 (0.08)
	40	35	−0.02 (0.46)	0.09	0.37 (0.06)
		50	0.04 (0.44)	0.07	0.38 (0.06)
	50	35	0.04 (0.42)	0.02	0.39 (0.05)
		50	0.14 (0.39)	<0.01	0.41 (0.04)
2 years	20	35	−0.29 (0.47)	0.92	0.30 (0.08)
		50	−0.27 (0.48)	0.90	0.33 (0.07)
	30	35	−0.19 (0.44)	0.65	0.33 (0.08)
		50	−0.16 (0.45)	0.58	0.35 (0.07)
	40	35	−0.11 (0.42)	0.33	0.36 (0.07)
		50	−0.08 (0.42)	0.22	0.38 (0.06)
	50	35	−0.06 (0.39)	0.12	0.38 (0.06)
		50	−0.02 (0.39)	0.07	0.40 (0.05)

Finally, if the population is considered to be totally isolated, heterozygosity would be expected to decrease from 0.47 (SD 0.01) to 0.30 (SD 0.08) over 20 years in the more pessimistic case, and from 0.47 (SD 0.01) to 0.41 (SD 0.04) in the more optimistic one (Table 3). Nevertheless, if it turns out to be only partially isolated, the immigration simulations revealed that this population could maintain at least 90% of its genetic variability (He≥0.42) if a minimum of three immigrants integrate the population every year, or if at least one immigration event occurs every two years, for the least and most favorable demographic parameters combinations respectively.

## Discussion

Our simulations suggest that the red squirrel population in the Parc de Sceaux is viable and could persist over the mid-term under certain conditions.

The population displays relatively high levels of genetic diversity and does not show significant levels of inbreeding although that does not exclude that a few individuals originated from related parents. It contains levels of genetic variability that are similar to those observed in the control population in the French Alps, both at the mitochondrial and nuclear level. It has a level of microsatellite variation similar to that of genetically diversified European populations (e.g., Italian Alps: mean He = 0.560, [37]; Jersey: mean He = 0.484, [38]) and higher than a genetically impoverished population (e.g., Wales: mean He = 0.242, [39]).

The population size is one of the crucial parameters on which the PVA is based. From the densities derived using the distance sampling, we estimated the size of the population as 104 to 124 individuals, a relatively small size, which may raise the question of the population persistence in the long term. It should be noted that large confidence intervals were associated with these densities. The distance sampling method recommends the detection of a minimum number of 60-80 individuals to achieve a suitable precision of density [40], [29] but we observed between 24 and 56 individuals per session. Our estimates from distance sampling should therefore be considered with caution. However, as recommended [41], [42] we used independent live trapping data to validate these estimates. Similar results were obtained from both methods, suggesting that the population size estimates based on distance sampling data are reliable.

Our results showed that both the age at which a female has its first litter and the juvenile survival rate strongly affect the population dynamics (Fig. 2). Under some conditions, i.e. juvenile survival (Sj) <25% or <40%, depending on the age at first litter, we obtained negative deterministic growth rates, indicating that the population is doomed to extinction, whether or not subjected to stochastic events. However, the population appears able to persist over time for optimistic scenarios (Sj>40% and first litter at 1 yr-old, or Sj = 50% and first litter at 2 yrs-old, Table 3) even when subjected to stochastic events. Under these last conditions, the probabilities of extinction allow classifying the population from “not endangered” to “viable”, for the most optimistic scenario, following the IUCN classification criteria (respectively, PE<20% within 20 years and PE<10% within 100 years; [35]).

Several elements suggest that the demographic parameters of the red squirrel population of the Parc de Sceaux are close to those of the optimistic scenarios. First, whether red squirrel females start reproducing at one or two-year-old largely depends on their body mass [43], [44]. The large amount of extra food brought to the squirrels by park visitors constitutes a relatively stable food supply throughout the year [23] and could therefore give young females the possibility to reproduce from the earliest age. Scenarios with an age at first litter of 1 year-old seem thus likely for the population of the Parc de Sceaux. This will have to be explored by continuing the trapping of squirrels to estimate the proportion of 1-year old females in estrus and/or lactating. Second, red squirrels survival directly depends on the availability of food [31], [32], and this is particularly so for juveniles [45]. The high feeding activity by the visitors of the park may therefore improve the survival of young individuals. In addition, causes of mortality such as predation [46] and road traffic [47], [48] probably have a low impact on the population of this park [23]. Under these circumstances, juvenile survival is likely to be high, close to 40-50%, as observed in other red squirrel populations [49], [50]. Finally, the annual estimates of the percentage of female reproducing in the Parc de Sceaux that we used to calibrate the scenarios (i.e., 35% or 50% at high density) might have been underestimated, since the trapping did not entirely overlap the two reproduction peaks of the red squirrel (first peak: February-April; second peak: May-August; [9], [23]). Our reproductive rate estimates are, nevertheless, similar to those encountered for other red squirrel populations in high density (0.36±0.18 [16]; 0.58±0.32 [50]). Therefore, we feel that the scenarios involving a “high-density reproductive rate” of 50% might be the most realistic for our population.

Significant loss of genetic diversity over 20 years is predicted by the PVA in all scenarios if the population is completely isolated. Such loss of genetic variability may decrease the likelihood of the population to persist into the future [51]. However, immigration simulations showed that the genetic diversity could be maintained on the long term if a minimum of 1 to 3 individuals per year settled in the population, depending on scenarios. These results are consistent with those of [49] indicating that more than a single immigration event per year would be needed to maintain genetic variation of partially isolated populations of red squirrels. A complete isolation of the Parc de Sceaux population seems unlikely since red squirrels are present in at least some of the surrounding forested areas (the closest is located 1 km from the park; [52]). We can reasonably suppose that immigration events, even episodic, occur in the population, since dispersing red squirrels may move over considerable distances, with a mean dispersal distances of 1014±925 m recorded in fragmented habitats [53], [54]. Hence, some juvenile or subadult immigrants should be able to cover the distance between the forested patches and settle in the Parc de Sceaux population. Furthermore, gardens, tree-rows and hedgerows around the park can be used by red squirrels to disperse in fragmented habitats [55], [12], [17]. If the existence of such flows between forested patches is verified, this urban population will therefore be able to maintain its genetic diversity level over time.

## Conclusions

The red squirrel population of Parc de Sceaux appears to be currently healthy, displaying a fairly high level of genetic diversity and no detectable inbreeding. Its relatively small size raises the question of its persistence, but several arguments suggest it could remain viable on the long term under certain conditions. Further research to (i) estimate the demographic parameters remaining uncertain (i.e., first age at breeding, juvenile survival and rate of breeding females), and (ii) investigate dispersal between populations, would nevertheless be required to verify this hypothesis. The present study suggests that the Parc de Sceaux is a suitable refuge for the red squirrels, and stresses the necessity of promoting and maintaining both ecological corridors and forested habitats in urban environments. This study also emphasizes the beneficial role of supplementary food on the viability of small populations, and asks the question whether or not to encourage such practices, as it has been suggested for a small island population of red squirrel in a similar case [56] (but see [57]). Studies of other urban parks would be necessary to reinforce our hypothesis of a positive role of such green refuges in the preservation of the red squirrel within urbanized areas.

## Supporting Information

Figure S1 **Histogram of perpendicular sighting distances.**
(DOC)

Table S1 **Selection of Distance Sampling models.**
(DOC)

Table S2 **Selection for density estimation using SECR models.**
(DOC)

Table S3 **PVA outcome with an initial population size of 50 individuals.**
(DOC)

File S1 **Details of microsatellite amplification and genotypes.**
(DOC)

File S2 **Details of population size estimation by Distance Sampling and Spatially Explicit Capture-Recapture methods.**
(DOC)

File S3 **Details of parameters chosen to set the PVA analysis.**
(DOC)
