# Supplementary material for: A Viable Population of the European Red Squirrel in an Urban Park
Source: PLoS One. 2014 Aug 15;9(8):e105111. doi: 10.1371/journal.pone.0105111 (PMC4134253; doi:10.1371/journal.pone.0105111)
Supplement: Figure S1 — Histogram of perpendicular sighting distances. (DOC) [file pone.0105111.s001.doc]

**Figure S1. Histogram of perpendicular sighting distances**

**
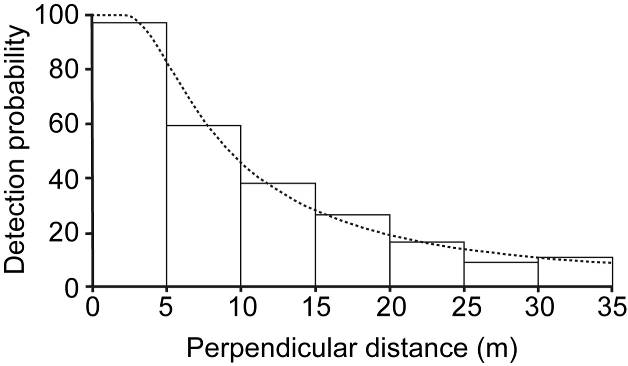
**

**Figure S1.** Histogram of perpendicular sighting distances
