# Supplementary material for: A Viable Population of the European Red Squirrel in an Urban Park
Source: PLoS One. 2014 Aug 15;9(8):e105111. doi: 10.1371/journal.pone.0105111 (PMC4134253; doi:10.1371/journal.pone.0105111)
Supplement: Table S1 — Selection of Distance Sampling models. (DOC) [file pone.0105111.s002.doc]

**Table S1. Selection of Distance Sampling models**

| Model (key function + adjustment term) | *K* | AIC | *δ*_AIC_ | χ*²* | *p* |
| --- | --- | --- | --- | --- | --- |
| Hazard rate + cosine | 2 | 562.09 | 0.00 | 1.00 | 0.91 |
| Half normal + cosine | 2 | 563.56 | 1.47 | 2.47 | 0.65 |
| Uniform + cosine | 3 | 565.24 | 3.15 | 2.15 | 0.54 |
| Uniform + simple polynomial | 3 | 568.15 | 6.06 | 5.16 | 0.16 |
| Half normal + hermite polynomial | 1 | 571.15 | 9.06 | 13.77 | 0.02 |
