# Supplementary material for: A Viable Population of the European Red Squirrel in an Urban Park
Source: PLoS One. 2014 Aug 15;9(8):e105111. doi: 10.1371/journal.pone.0105111 (PMC4134253; doi:10.1371/journal.pone.0105111)
Supplement: Table S2 — Selection for density estimation using SECR models. (DOC) [file pone.0105111.s003.doc]

**Table S2. Selection for density estimation using SECR models**

| Model | *K* | AIC_c_ | *δ*_AICc_ | Weight |
| --- | --- | --- | --- | --- |
| D~session*^a^*, g0~b, σ~b*^b^* | 9 | 2302.01 | 0.00 | 0.97 |
| D~session, g0~1, σ~1 | 7 | 2309.94 | 7.93 | 0.02 |
| D~session, g0~1, σ~b | 8 | 2311.80 | 9.79 | <0.01 |
| D~session, g0~b, σ~1 | 8 | 2311.98 | 9.98 | <0.01 |

^a^ Density estimate realized by session

^b^ Model tested for the influence of a learned response to trap on the detection probability (g0) and on the movement scale (σ)
