# Supplementary material for: A Viable Population of the European Red Squirrel in an Urban Park
Source: PLoS One. 2014 Aug 15;9(8):e105111. doi: 10.1371/journal.pone.0105111 (PMC4134253; doi:10.1371/journal.pone.0105111)
Supplement: Table S3 — PVA outcome with an initial population size of 50 individuals. (DOC) [file pone.0105111.s004.doc]

**Table S3. PVA outcome with an initial population size of 50 individuals**

| **Scenarios** |  |  |  |  | **Stochastic growth rate (SD)** | **Probability of extinction** | **Expected heterozygosity (SD)** |
| --- | --- | --- | --- | --- | --- | --- | --- |
|  |  | **Rate (%) of** |  |  |  |  |  |
| **Age at first litter** |  | **Juvenile survival** | **Breeding females at high density** |  |  |  |  |
| 1 year |  | 20 | 35 |  | -0.23 (0.50) | 0.86 | 0.29 (0.08) |
|  |  |  | 50 |  | -0.19 (0.55) | 0.83 | 0.31 (0.09) |
|  |  | 30 | 35 |  | -0.09 (0.52) | 0.44 | 0.33 (0.08) |
|  |  |  | 50 |  | -0.05 (0.51) | 0.39 | 0.35 (0.08) |
|  |  | 40 | 35 |  | 0.01 (0.46) | 0.10 | 0.37 (0.07) |
|  |  |  | 50 |  | 0.06 (0.45) | 0.08 | 0.38 (0.06) |
|  |  | 50 | 35 |  | 0.07 (0.42) | 0.02 | 0.39 (0.05) |
|  |  |  | 50 |  | 0.14 (0.40) | 0.01 | 0.40 (0.05) |
| 2 years |  | 20 | 35 |  | -0.26 (0.47) | 0.96 | 0.32 (0.07) |
|  |  |  | 50 |  | -0.24 (0.48) | 0.94 | 0.32 (0.08) |
|  |  | 30 | 35 |  | -0.16 (0.46) | 0.73 | 0.32 (0.07) |
|  |  |  | 50 |  | -0.15 (0.46) | 0.68 | 0.33 (0.07) |
|  |  | 40 | 35 |  | -0.09 (0.43) | 0.40 | 0.34 (0.07) |
|  |  |  | 50 |  | -0.07 (0.42) | 0.34 | 0.36 (0.07) |
|  |  | 50 | 35 |  | -0.04 (0.41) | 0.18 | 0.37 (0.06) |
|  |  |  | 50 |  | -0.01 (0.40) | 0.15 | 0.38 (0.06) |
