# Supplementary material for: A Viable Population of the European Red Squirrel in an Urban Park
Source: PLoS One. 2014 Aug 15;9(8):e105111. doi: 10.1371/journal.pone.0105111 (PMC4134253; doi:10.1371/journal.pone.0105111)
Supplement: File S1 — Details of microsatellite amplification and genotypes. (DOC) [file pone.0105111.s005.doc]

**File S1.** **Details of microsatellite amplification and Genotypes**

Individual samples were genotyped at 13 microsatellites loci previously developed for *S. vulgaris*: Scv1, Scv3, Scv6, Scv8, Scv9, Scv12, Scv13, Scv14, Scv15 (Hale *et al.*, 2001) and Rsu3, Rsu4, Rsu5, Rsu6 (Todd, 2000). Amplification was performed in 15µL of PCR reaction containing 1µl of DNA solution, 0.2 µM of each primer, 400µM of dNTP, 1 x *Taq* reaction buffer and 0.5 units/µl of Taq Polymerase (Qiagen). Thermocycles conditions were as follow: an initial denaturation step of 1 min at 94°C, followed by 35 thermocycles of 94°C for 30 s, 53°C for 30s, 72°C for 1 min and a final extension at 72°C for 7 min. The PCR products were mixed with an internal size standard and analyzed using an ABI 3700 capillary DNA sequencer (Applied Biosystems) in the Plateforme Gentyane (INRA).

Hale, M.L., Bevan, R. & Wolff, K. (2001). New polymorphic microsatellite markers for the red squirrel (*Sciurus vulgaris*) and their applicability to the grey squirrel (*S. carolinensis*). *Molecular Ecology Notes* **1**, 47–49.

Todd, R. (2000). Microsatellite loci in the Eurasian red squirrel, *Sciurus vulgaris* L. *Mol. Ecol.* **9**, 2165–2166.

***GENEPOP format microsatellite dataset***

Rsu3

Rsu4

Rsu5

Rsu6

Scv6

Scv8

Scv1

Scv12

Scv13

Scv14

Scv15

Scv3

Scv9

POP

37 , 163165 262262 137137 119126 194197 199201 177181 199201 171181 194194 181181 211217 197199

87 , 000000 000000 000000 126126 000000 000000 000000 000000 000000 194194 000000 205220 000000

88 , 163163 262262 137137 119126 194194 199199 000000 199199 000000 194194 181181 184207 197199

89 , 163163 000000 137137 119119 194197 199206 169179 197199 181181 194194 181181 188203 197199

90 , 163165 000000 137137 126126 000000 000000 179181 000000 000000 194194 181181 203203 000000

91 , 165165 000000 000000 126126 000000 000000 000000 201201 000000 194194 181181 000000 000000

92 , 163167 262270 137137 126126 194199 199199 169177 197197 181181 194194 181181 186188 195199

93 , 000000 000000 000000 000000 000000 000000 000000 000000 000000 000000 000000 184184 000000

94 , 165165 262262 137144 119126 197203 199199 000000 000000 000000 194194 181181 000000 000000

95 , 163163 266270 137137 126126 194197 199199 000000 201201 000000 194194 181181 203205 000000

96 , 163163 000000 000000 126126 000000 000000 000000 199201 000000 194194 181181 203205 000000

97 , 163163 000000 137137 126126 194197 199199 000000 197201 000000 194194 181181 203205 000000

98 , 165165 000000 000000 126126 194194 000000 000000 199199 171183 000000 181181 209209 195195

99 , 000000 000000 000000 000000 000000 000000 000000 000000 000000 000000 000000 000000 000000

1000 , 165165 000000 000000 126126 000000 000000 000000 000000 181181 194194 181185 203205 000000

1001 , 163165 262262 000000 126126 194197 000000 177179 000000 181181 194194 000000 000000 000000

1002 , 163165 000000 000000 126126 194194 000000 177177 000000 000000 194194 179181 000000 000000

1003 , 163165 000000 000000 126126 194199 199199 000000 199199 000000 194194 000000 205220 000000

1004 , 163163 000000 000000 126126 000000 000000 000000 000000 181181 194194 181181 184188 000000

A , 163165 262262 137137 119126 194197 199201 177181 000000 171181 194194 181181 211217 000000

B , 163165 000000 000000 126126 197203 000000 177179 000000 000000 000000 181181 184184 000000

C , 163165 262282 137137 126126 194199 199199 177179 199199 171181 194194 181181 205209 195199

D_Savoie , 163163 000000 000000 126126 194197 000000 000000 000000 000000 194194 181181 203205 000000

POP

10167 , 163165 000000 000000 119126 000000 000000 177177 199199 171171 194196 181181 207207 195199

1085 , 163165 274274 000000 119126 194194 203203 177177 000000 171171 194196 000000 209215 000000

1151 , 163165 262282 137137 119126 000000 199203 177181 199201 181183 194194 181181 209213 199199

1240 , 163163 262274 000000 119126 194197 203203 177177 199199 171171 196196 181181 213215 000000

1267 , 163165 274274 122137 119126 194201 203203 177181 199199 171181 194196 181181 213222 195199

1279 , 163163 274282 122137 119126 194197 203203 177177 000000 000000 000000 000000 184209 000000

146 , 163165 274274 000000 119119 194197 203203 177177 197199 171171 196198 181181 213215 195195

1473 , 163163 274274 137137 119126 194194 203203 177181 199199 171171 194196 181181 184222 195199

1693 , 163165 000000 000000 119126 194197 000000 177181 000000 000000 198198 000000 209209 000000

1999 , 163165 000000 122122 119126 194197 203203 177181 197199 171171 194194 181181 186188 199199

2151 , 163165 274274 122122 119119 194194 203203 177181 199199 171171 196196 181181 184209 195199

267 , 163163 266274 122137 126126 194197 201203 177183 197199 171171 194194 181181 186207 000000

2736 , 163165 274274 122137 126126 194197 203203 000000 199199 171171 194196 181181 203209 195199

293 , 163163 262274 122137 119119 194194 199203 177179 197199 171171 194198 181181 209215 199199

3052 , 163163 274282 122137 119119 194194 203203 177181 199199 171171 196198 181181 209209 195197

3289 , 163165 262274 122137 119126 194201 199203 177181 000000 000000 000000 000000 188188 000000

3511 , 165165 274274 137137 119126 194199 203203 177181 199199 171183 198198 181181 215215 000000

355 , 163163 262266 137137 119126 194194 201203 177183 199199 171171 194196 181181 186213 195199

3608 , 163165 274274 000000 119126 197197 203203 177177 000000 171171 194198 181181 000000 000000

3767 , 165165 000000 137137 119126 000000 000000 177177 199199 181183 196196 181181 184188 195195

3976 , 163165 274274 000000 126126 194199 203203 177177 000000 000000 196196 000000 000000 000000

4128 , 165165 274274 122137 119126 194199 203203 177177 199199 171183 194194 181181 188213 195195

4244 , 000000 000000 000000 000000 000000 000000 177181 000000 000000 196196 000000 184184 000000

425 , 165165 274274 122137 119126 194199 203203 177181 199199 171171 196198 000000 184184 000000

442 , 163165 274282 000000 119119 194199 203203 177181 199199 171183 194198 181181 184186 195199

4609 , 163165 274274 000000 126126 194199 199203 177181 199201 171183 194196 181181 209215 195195

485 , 163165 262274 122137 119119 197199 203203 177177 199199 171183 194196 181181 209215 195199

5005 , 163165 274278 000000 119126 194197 000000 177177 199199 171171 196196 181181 213215 000000

5119 , 163163 000000 000000 119126 197199 000000 177177 000000 000000 196196 000000 000000 000000

5239 , 163163 274282 122137 119119 194199 199203 177181 199199 171183 194196 181181 209209 199199

5240 , 165165 274282 137137 119119 194199 199203 177181 199199 181183 194198 181181 184213 195199

5876 , 163165 274278 137137 119126 194199 000000 177177 199199 171183 194194 181181 186209 195195

5890 , 163163 274274 137137 119126 194201 203203 177181 199199 000000 196198 181181 184209 195199

591 , 163163 000000 000000 119119 194197 000000 177181 000000 000000 194196 000000 000000 000000

5917 , 163165 274274 137137 119126 194194 000000 177177 000000 171171 196196 000000 000000 000000

6051 , 000000 000000 000000 119126 000000 199203 177177 197199 171181 198198 181181 209213 195195

6681 , 000000 000000 000000 000000 000000 000000 177181 199199 171183 196196 000000 209215 195195

6828 , 165165 262274 137137 126126 194197 203203 177181 199199 171171 196198 181181 209215 195199

6877 , 163163 274278 000000 126126 194197 000000 177177 199199 171171 196198 181181 213215 000000

6913 , 165165 000000 122137 119126 194199 203203 177177 199199 000000 194194 000000 000000 000000

6979 , 163165 274282 122137 119126 194197 199203 177183 199199 171171 198198 181181 213222 195199

7064 , 163163 000000 000000 119119 194194 000000 177177 199199 171171 196196 181181 000000 195199

7065 , 163163 274274 122137 119126 194199 203203 177177 199199 171183 196196 181181 184213 195195

7208 , 163165 274274 000000 119119 194197 191203 177181 199199 000000 196198 000000 213213 195195

736 , 163163 274282 137137 119126 194194 203203 177181 197199 171171 196198 181181 209209 000000

7425 , 163165 274282 137137 119119 194194 203203 177181 199199 171171 000000 181181 184209 195195

7440 , 163163 274274 122137 119126 194194 203203 177177 199199 171171 194196 181181 184203 197199

7626 , 000000 000000 000000 119126 000000 000000 177177 199199 171183 194196 181181 188215 195195

7660 , 163163 274274 000000 119126 000000 000000 177177 199199 171171 194196 181181 188215 000000

7753 , 165165 274282 122137 126126 194194 199203 177177 199199 171181 194198 181181 213215 195199

8058 , 000000 274278 000000 126126 197197 203203 177177 000000 000000 196196 181181 184184 195195

8100 , 163163 274274 137137 119122 199201 203203 177177 197199 181183 196196 181181 209209 199199

8178 , 000000 000000 000000 000000 000000 000000 177183 199199 171183 196196 000000 209213 000000

863 , 163163 274274 122137 119126 194194 203203 177181 197199 171171 198198 181181 209209 199199

8876 , 163163 000000 000000 119119 000000 000000 177181 197199 171171 194196 181181 209209 195199

8877 , 163165 274282 000000 122122 194197 203203 177177 199199 171171 194196 181181 184217 195199

8906 , 165165 274274 000000 126126 194197 203203 177177 199199 171171 196196 181181 213213 195195

9023 , 163163 000000 000000 119119 000000 000000 177177 199199 171183 194196 181181 209209 195199

9041 , 163163 000000 000000 119126 194197 000000 000000 199199 171181 196196 181181 186209 199199

9051 , 000000 000000 137137 119126 000000 000000 177181 199199 171181 194196 181181 213222 195199

9320 , 163163 000000 122137 119126 194197 203203 000000 000000 000000 000000 000000 000000 000000

9393 , 163163 000000 000000 119126 000000 000000 177181 197199 171171 194198 181181 209209 195195

9533 , 163165 274282 137137 119126 194201 203203 177181 199199 171181 196198 181181 213222 195199

9761 , 163165 000000 000000 119119 000000 000000 000000 000000 000000 194196 000000 000000 000000

9969 , 165165 274274 000000 126126 194199 203203 177177 199199 000000 194194 181181 188215 195199

b149 , 163163 262274 137137 126126 194197 203203 177177 199201 171171 196196 181181 209209 199199

b887 , 163163 262274 137137 126126 194194 203203 177177 197199 171171 196196 181181 186213 195199

b980_Sceaux , 163163 274274 137137 126126 194199 203203 177177 199199 171183 194194 181181 209209 195199
