# Supplementary material for: A Viable Population of the European Red Squirrel in an Urban Park
Source: PLoS One. 2014 Aug 15;9(8):e105111. doi: 10.1371/journal.pone.0105111 (PMC4134253; doi:10.1371/journal.pone.0105111)
Supplement: File S2 — Details of population size estimation by Distance Sampling and Spatially Explicit Capture-Recapture methods. (DOC) [file pone.0105111.s006.doc]

**File S2. Details of population size estimation by Distance Sampling and Spatially Explicit Capture-Recapture methods**

**Distance Sampling**

Eighteen permanent line-transects, ranging from 187 to 408 meters were randomly set on the park, covering a total distance of approximately 5 km (Fig. 1– in the text). Two sessions were performed, one in November-December 2012 and one in March 2013 in absence of tree leaves. Transects were walked by the same observer (CR) between 7:00 and 11:00 (UT), when squirrels are the most active, and the perpendicular distance from the line to all individuals detected was recorded. Each transect line was walked once daily, 4 times per session. Observations from temporal replicates were pooled by transect and treated as a single sample (sample size=18) for each session (Buckland *et al.*, 2010). Data were analyzed using DISTANCE 6.0 (Thomas *et al.*, 2010). A truncation of 5% of the greatest perpendicular distances was applied to the dataset to delete outliers and improve subsequent model-fitting (Buckland *et al.*, 1993). As the visibility did not differ across areas during the study periods, the detection function was generated by pooling all the observations from both parts of the park. This function was modeled using the uniform, half-normal and hazard-rate key function, combined with the cosine, polynomial and hermite polynomial adjustment terms (Buckland *et al.*, 2001). Five models suggested by Buckland *et al.* (2001) were tested and best density estimates were inferred from model selection based on minimum Akaike’s Information Criterion score (AIC) and on Chi-square goodness-of-fit tests.

**Data used in Distance Sampling analyses**

**Data set**: **Site A/Site B** = Western / Eastern areas respectively; **nov/fev** = session month (November/December 2012, February/March 2013, see Table 1 in the text); **Line-transect number** 1-18 (see Fig. 1 in the text); **Cumulative line-transect length** over the 4 temporal replications (in m); **Perpendicular distance** (in m) to the line-transect of an observation of red squirrel.

Example: *“Site Anov;Line 10;748;9” An individual was seen in the Western area during the November/December session in 2012 at 9m perpendicularly to the line 10, which corresponds to a sampling effort of 748m long over the 4 replications.*

Site Anov;Line 10;748;9

Site Anov;Line 10;748;10

Site Anov;Line 10;748;34

Site Anov;Line 11;1476;1

Site Anov;Line 11;1476;2

Site Anov;Line 11;1476;6

Site Anov;Line 12;1504;0

Site Anov;Line 12;1504;0

Site Anov;Line 12;1504;0

Site Anov;Line 12;1504;0

Site Anov;Line 12;1504;1

Site Anov;Line 12;1504;2

Site Anov;Line 12;1504;6

Site Anov;Line 12;1504;6

Site Anov;Line 12;1504;13

Site Anov;Line 12;1504;14

Site Anov;Line 12;1504;14

Site Anov;Line 12;1504;18

Site Anov;Line 12;1504;18

Site Anov;Line 12;1504;23

Site Anov;Line 13;952;0

Site Anov;Line 13;952;1

Site Anov;Line 13;952;1

Site Anov;Line 13;952;3

Site Anov;Line 13;952;4

Site Anov;Line 13;952;4

Site Anov;Line 13;952;5

Site Anov;Line 13;952;12

Site Anov;Line 13;952;17

Site Anov;Line 13;952;37

Site Anov;Line 14;1148;

Site Anov;Line 15;1632;3

Site Anov;Line 16;844;1,5

Site Anov;Line 16;844;10

Site Anov;Line 17;1452;4

Site Anov;Line 18;1272;8

Site Anov;Line 18;1272;9

Site Afev;Line 10;748;5

Site Afev;Line 11;1476;0

Site Afev;Line 11;1476;4

Site Afev;Line 11;1476;34

Site Afev;Line 12;1504;0

Site Afev;Line 12;1504;7

Site Afev;Line 12;1504;9

Site Afev;Line 12;1504;11

Site Afev;Line 12;1504;15

Site Afev;Line 13;952;0

Site Afev;Line 13;952;0

Site Afev;Line 13;952;0

Site Afev;Line 13;952;3

Site Afev;Line 13;952;3

Site Afev;Line 13;952;8

Site Afev;Line 13;952;9

Site Afev;Line 13;952;16

Site Afev;Line 13;952;29

Site Afev;Line 13;952;30

Site Afev;Line 13;952;38

Site Afev;Line 13;952;38

Site Afev;Line 13;952;39

Site Afev;Line 13;952;40

Site Afev;Line 14;1148;

Site Afev;Line 15;1632;29

Site Afev;Line 16;844;9

Site Afev;Line 16;844;11

Site Afev;Line 16;844;14

Site Afev;Line 16;844;22

Site Afev;Line 17;1452;

Site Afev;Line 18;1272;

Site Bnov;Line 1;788;5

Site Bnov;Line 1;788;6

Site Bnov;Line 1;788;6

Site Bnov;Line 1;788;9

Site Bnov;Line 1;788;15

Site Bnov;Line 1;788;17

Site Bnov;Line 1;788;18

Site Bnov;Line 1;788;22

Site Bnov;Line 2;968;0

Site Bnov;Line 2;968;1

Site Bnov;Line 2;968;4

Site Bnov;Line 3;1028;

Site Bnov;Line 4;1024;3

Site Bnov;Line 4;1024;12

Site Bnov;Line 4;1024;16

Site Bnov;Line 4;1024;22

Site Bnov;Line 5;1252;0

Site Bnov;Line 5;1252;13

Site Bnov;Line 5;1252;31

Site Bnov;Line 6;1112;18

Site Bnov;Line 6;1112;20

Site Bnov;Line 7;1156;0

Site Bnov;Line 7;1156;12

Site Bnov;Line 7;1156;13

Site Bnov;Line 7;1156;16

Site Bnov;Line 8;840;0

Site Bnov;Line 8;840;0

Site Bnov;Line 8;840;0

Site Bnov;Line 8;840;1

Site Bnov;Line 8;840;2

Site Bnov;Line 8;840;6

Site Bnov;Line 8;840;6

Site Bnov;Line 8;840;8

Site Bnov;Line 8;840;9

Site Bnov;Line 8;840;9

Site Bnov;Line 8;840;10

Site Bnov;Line 8;840;10

Site Bnov;Line 8;840;11

Site Bnov;Line 8;840;18

Site Bnov;Line 8;840;22

Site Bnov;Line 8;840;24

Site Bnov;Line 8;840;30

Site Bnov;Line 9;832;0

Site Bnov;Line 9;832;0

Site Bnov;Line 9;832;0

Site Bnov;Line 9;832;1

Site Bnov;Line 9;832;1

Site Bnov;Line 9;832;2

Site Bnov;Line 9;832;3

Site Bnov;Line 9;832;3

Site Bnov;Line 9;832;6

Site Bnov;Line 9;832;9

Site Bnov;Line 9;832;10

Site Bnov;Line 9;832;12

Site Bnov;Line 9;832;12

Site Bnov;Line 9;832;20

Site Bnov;Line 9;832;35

Site Bnov;Line 9;832;36

Site Bnov;Line 9;832;44

Site Bfev;Line 1;788;

Site Bfev;Line 2;968;0

Site Bfev;Line 2;968;0

Site Bfev;Line 2;968;3

Site Bfev;Line 2;968;4

Site Bfev;Line 2;968;6

Site Bfev;Line 2;968;8

Site Bfev;Line 2;968;10

Site Bfev;Line 2;968;10

Site Bfev;Line 2;968;13

Site Bfev;Line 3;1028;4

Site Bfev;Line 4;1024;6

Site Bfev;Line 5;1252;0

Site Bfev;Line 5;1252;1

Site Bfev;Line 5;1252;3

Site Bfev;Line 5;1252;5

Site Bfev;Line 5;1252;10

Site Bfev;Line 5;1252;17

Site Bfev;Line 5;1252;27

Site Bfev;Line 6;1112;0

Site Bfev;Line 6;1112;5

Site Bfev;Line 6;1112;6

Site Bfev;Line 6;1112;10

Site Bfev;Line 6;1112;15

Site Bfev;Line 6;1112;19

Site Bfev;Line 6;1112;20

Site Bfev;Line 6;1112;34

Site Bfev;Line 7;1156;5

Site Bfev;Line 7;1156;11

Site Bfev;Line 7;1156;12

Site Bfev;Line 7;1156;16

Site Bfev;Line 7;1156;21

Site Bfev;Line 8;840;0

Site Bfev;Line 8;840;2

Site Bfev;Line 8;840;6

Site Bfev;Line 8;840;7

Site Bfev;Line 8;840;9

Site Bfev;Line 8;840;11

Site Bfev;Line 8;840;13

Site Bfev;Line 8;840;13

Site Bfev;Line 8;840;21

Site Bfev;Line 8;840;23

Site Bfev;Line 8;840;23

Site Bfev;Line 8;840;24

Site Bfev;Line 8;840;31

Site Bfev;Line 8;840;39

Site Bfev;Line 9;832;0

Site Bfev;Line 9;832;0

Site Bfev;Line 9;832;2

Site Bfev;Line 9;832;6

Site Bfev;Line 9;832;7

Site Bfev;Line 9;832;12

Site Bfev;Line 9;832;15

Site Bfev;Line 9;832;18

Site Bfev;Line 9;832;28

Site Bfev;Line 9;832;31

**SECR method**

In the eastern part, the presence of protected areas with partially restricted access allowed to estimate the density of red squirrels using live trapping (Dozières, 2012). CMR sessions were carried out twice, in October 2012 and in February 2013. Each session consisted of 5 consecutive trapping days using 41 geo-localized live traps baited with walnuts (Fig. 1 – in the text). Traps were opened in the morning and closed in the end of afternoon and checked three times a day. Density estimate was performed using spatially explicit capture-recapture models with the maximum likelihood method (Efford, 2004; Borchers & Efford, 2008) implemented in the package “secr” in R (Efford, 2012). Home-range centres were assumed to be Poisson distributed, and the detection function followed a half-normal curve, where capture probability decreased with the distance to the trap. The spatial boundary strip was set at 200 m and the spacing for the integration mesh of the ML estimator was set to 36x36 points, matching the contour of the trapping grid. A non-habitat mask was applied to the grid, excluding lawns and water channels areas. We ran models with constant parameters, and tested for an influence of learned response at first capture (noted ‘b’) on each parameter of the model, i.e., detection probability noted ‘g(0)’ and movement scale noted ‘σ’. The best model was selected using difference in the second order AIC (i.e., AICc). A conditional likelihood incorporating the different trapping sessions was used to derive estimate of densities.

**Data used in Spatially Explicit Capture Recapture analyses**

**Traps coordinates**

Trap X-coord Y-coord

1 597252 2419350

2 597214 2419351

3 597239 2419311

4 597254 2419239

5 597237 2419202

6 597314 2419271

7 597338 2419164

8 597356 2419120

9 597387 2419066

10 597291 2419148

11 597286 2419118

12 597362 2419053

13 597376 2419015

14 597464 2419129

15 597513 2419073

16 597485 2419036

17 597531 2419008

18 597559 2418974

19 597575 2418815

20 597577 2418876

21 597411 2418788

22 597394 2418777

23 597375 2418791

24 597362 2418766

25 597347 2418753

26 597155 2419361

27 597143 2419405

28 597188 2419365

29 597343 2419223

30 597347 2418979

31 597312 2419003

32 597280 2419032

37 597298 2419105

38 597553 2419044

39 597583 2418951

40 597632 2418889

41 597640 2418865

42 597656 2418836

43 597490 2418581

44 597484 2418605

45 597495 2418653

**Coordinates for non-habitat mask**

X-coord Y-coord

597283 2418281

597611 2418347

597779 2418618

597759 2418754

597711 2418754

597637 2418602

597360 2418534

597298 2418836

597405 2418862

597415 2418824

597465 2418791

597486 2418747

597514 2418751

597513 2418801

597546 2418852

597519 2418965

597481 2418996

597413 2418977

597392 2418934

597398 2418892

597292 2418871

597200 2419210

597171 2419239

597146 2419337

597070 2419319

597283 2418281

596974 2419410

597187 2419450

597211 2419440

597488 2419501

597435 2419409

597431 2419309

597482 2419222

597629 2419256

597548 2419655

596941 2419527

596974 2419410

**Squirrels capture histories**

Se: sessions

(6=October 2012,

7=February 2013);

ID: squirrel identity;

Oc: Day occasions;

Traps: Trap identity.

Se ID Oc Traps

6 14 1 3

6 19 1 25

6 22 1 41

6 23 1 40

6 24 1 17

6 100 1 17

6 577 1 28

6 585 1 5

6 588 1 18

6 591 1 1

6 592 1 4

6 596 1 14

6 702 1 28

6 705 1 12

6 713 1 13

6 819 1 10

6 841 1 2

6 844 1 1

6 850 1 15

6 889 1 31

6 892 1 16

6 897 1 39

6 24 2 18

6 98 2 4

6 507 2 37

6 508 2 8

6 591 2 1

6 592 2 29

6 597 2 14

6 701 2 39

6 705 2 30

6 711 2 7

6 834 2 18

6 888 2 5

6 898 2 38

6 14 3 1

6 16 3 25

6 21 3 23

6 22 3 19

6 98 3 6

6 507 3 10

6 508 3 13

6 588 3 9

6 592 3 10

6 702 3 3

6 713 3 31

6 845 3 31

6 887 3 29

6 14 4 4

6 21 4 25

6 22 4 40

6 23 4 41

6 24 4 38

6 98 4 4

6 100 4 38

6 507 4 11

6 585 4 5

6 588 4 17

6 592 4 6

6 702 4 26

6 705 4 10

6 711 4 14

6 713 4 9

6 841 4 28

6 844 4 3

6 889 4 13

6 890 4 16

6 993 4 6

6 17 5 25

6 22 5 19

6 23 5 40

6 24 5 39

6 98 5 4

6 507 5 8

6 508 5 12

6 585 5 5

6 588 5 18

6 592 5 5

6 701 5 38

6 705 5 31

6 711 5 11

6 713 5 30

6 819 5 9

6 841 5 26

6 850 5 16

6 887 5 6

6 889 5 12

7 16 1 22

7 22 1 20

7 41 1 27

7 98 1 6

7 507 1 11

7 508 1 8

7 585 1 29

7 809 1 8

7 833 1 29

7 856 1 40

7 980 1 41

7 16 2 24

7 23 2 19

7 98 2 29

7 100 2 38

7 508 2 9

7 585 2 6

7 588 2 18

7 705 2 10

7 711 2 12

7 840 2 27

7 841 2 1

7 887 2 4

7 888 2 5

7 902 2 41

7 904 2 24

7 977 2 45

7 980 2 42

7 19 3 25

7 24 3 39

7 98 3 4

7 585 3 4

7 596 3 14

7 597 3 16

7 705 3 37

7 711 3 11

7 828 3 6

7 836 3 6

7 850 3 14

7 889 3 31

7 980 3 40

7 22 4 41

7 23 4 40

7 24 4 39

7 507 4 7

7 585 4 3

7 705 4 7

7 711 4 30

7 833 4 3

7 840 4 2

7 841 4 28

7 847 4 25

7 850 4 15

7 888 4 6

7 22 5 20

7 98 5 6

7 588 5 39

7 711 5 10

7 719 5 14

7 828 5 6

7 833 5 27

7 850 5 16

7 887 5 29

7 889 5 12

**References for File S2**

Borchers, D.L. & Efford, M.G. (2008). Spatially explicit maximum likelihood methods for capture–recapture studies. *Biometrics* **64**, 377–385.

Buckland, S.T., Anderson, D.R., Burnham, K.P. & Laake, J.L. (1993). *Distance Sampling: estimating abundance of biological populations*, Chapman and Hall, London, reprinted 1999 by RUWPA, University of St. Andrews, Scotland.

Buckland, S.T., Plumptre, A.J., Thomas, L. & Rexstad, E.A. (2010). Design and analysis of line transect surveys for primates. *Int. J. Primatol.* **31**, 833–847.

Buckland, S.T., Anderson, D.R., Burnham, K.P. & Laake, J.L., Borchers, D.L. & Thomas, L. (2001). *Introduction to distance sampling*. Oxford University Press, Oxford.

Dozières, A. (2012). *Conservation de l'écureuil roux en France : de l'état des populations aux enjeux liés à l'introduction de l'écureuil à ventre rouge.* PhD thesis, Muséum National d’Histoire Naturelle, Paris.

Efford, M.G. (2004). Density estimation in live-trapping studies. *Oikos* **106**, 598–610.

Efford, M.G. (2012). *secr: spatially explicit capture-recapture models. R package version 2.3.2.* Available from <http://CRAN.R-project.org/package=secr> (accessed December 2011).

Thomas, L., Buckland, S.T., Rexstad, E.A., Laake, J.L., Strindberg, S., Hedley, S.L., Bishop, J.R.B., Marques, T.A. & Burnham, K.P. (2010).  Distance software: design and analysis of distance sampling surveys for estimating population size.  *J. Appl. Ecol.* **47**, 5–14.
