# Supplementary material for: A Viable Population of the European Red Squirrel in an Urban Park
Source: PLoS One. 2014 Aug 15;9(8):e105111. doi: 10.1371/journal.pone.0105111 (PMC4134253; doi:10.1371/journal.pone.0105111)
Supplement: File S3 — Details of parameters chosen to set the PVA analysis. (DOC) [file pone.0105111.s007.doc]

**File S3. Details of parameters chosen to set the PVA analysis**

Red squirrels are able to reproduce from the age of 1 year-old (Lurz, Gurnell & Magris,, 2005) but the average observed age at first litter recorded for this species is often closer to 2 years-old (Wauters & Dhondt, 1995). We used both ages in our models, respectively as an optimistic and moderate estimate of the age at which squirrel of this population have their first litter.

Density-dependent reproduction is known to occur in red squirrel (Wauters & Lens, 1995; Wauters *et al.,* 2004). Based on the number of adult females seen in estrus (in February) or lactating (in October) during trapping sessions, we estimated the proportion of female breeding in the population to be 35% in 2011 and 50% in 2012 (Unpublished data). Density-dependent reproduction was therefore integrated in the model assuming that either 35% or 50% of the females would be breeding (once or twice in a year) at high density (n=K; present study), and 70% at low density (n<K; see Wauters *et al.*, 2004).

No data were available for juvenile survival (Dozières, 2012). Pessimistic to optimistic scenarios were realized by varying juvenile survival between 20% and 50% (Gurnell, 1983; 1987; Wauters *et al.*, 1994).

The initial population size (n) was set based on results of the population size estimates presented above. As the red squirrel population in Sceaux reaches the highest densities found for this species (Dozières, 2012), we considered the population has reached its maximum size. Hence we set the carrying capacity (K) to a minimum value, corresponding to the current population size.

Dozières, A. (2012). *Conservation de l'écureuil roux en France : de l'état des populations aux enjeux liés à l'introduction de l'écureuil à ventre rouge.* PhD thesis, Muséum National d’Histoire Naturelle, Paris.

Gurnell, J. (1983). Squirrel numbers and the abundance of tree seeds. *Mammal Rev.* **13**, 133–148.

Gurnell, J. (1987). *The natural history of squirrels*. Christopher Helm, London.

Lurz, P.W.W., Gurnell, J. & Magris, L. (2005). *Sciurus vulgaris*. *Mamm. Species* **769**, 1–10.

Wauters, L.A., & Dhondt, A.A. (1995). Lifetime reproductive success and its correlates in female Eurasian red squirrels. *Oikos* **72**, 402–410.

Wauters, L.A. & Lens, L. (1995). Effects of food availability and density on red squirrel *(Sciurus vulgaris)* reproduction. *Ecology* **76**, 2460–2469.

Wauters, L.A., Matthysen, E., Adriasen, F. & Tosi, G. (2004). Within-sex density dependence and population dynamics of red squirrels *Sciurus vulgaris*. *J. Anim. Ecol.* **73**, 11–25.

Wauters, L.A., Hutchinson, Y., Parkin, D.T. & Dhondt, A.A. (1994). The effects of habitat fragmentation on demography and on the loss of genetic variation in the red squirrel. *Proc. Roy. Soc. B—Biol. Sci.* **255**, 107–111.
